# Impact of altered phosphorylation on loss of function of juvenile Parkinsonism–associated genetic variants of the E3 ligase parkin

**DOI:** 10.1074/jbc.RA117.000605

**Published:** 2018-03-12

**Authors:** Jacob D. Aguirre, Karen M. Dunkerley, Rica Lam, Michele Rusal, Gary S. Shaw

**Affiliations:** From the Department of Biochemistry, University of Western Ontario, London, Ontario N6A 5C1, Canada

**Keywords:** parkin, PTEN-induced putative kinase 1 (PINK1), protein structure, protein folding, protein–protein interaction, ubiquitylation (ubiquitination), Parkinson's disease (autosomal recessive, early onset) 7 (PARK7)

## Abstract

Autosomal recessive juvenile Parkinsonism (ARJP) is an inherited neurodegenerative disease in which 50% of affected individuals harbor mutations in the gene encoding the E3 ligase parkin. Parkin regulates the mitochondrial recycling pathway, which is induced by oxidative stress. In its native state, parkin is auto-inhibited by its N-terminal ubiquitin-like (Ubl) domain, which blocks the binding site for an incoming E2∼ubiquitin conjugate, needed for parkin's ubiquitination activity. Parkin is activated via phosphorylation of Ser-65 in its Ubl domain by PTEN-induced putative kinase 1 (PINK1) and a ubiquitin molecule phosphorylated at a position equivalent to Ser-65 in parkin. Here we have examined the underlying molecular mechanism of phosphorylation of parkin's Ubl domain carrying ARJP-associated substitutions and how altered phosphorylation modulates parkin activation and ubiquitination. We found that three substitutions in the Ubl domain (G12R, R33Q, and R42P) significantly decrease PINK1's ability to phosphorylate the Ubl domain. We noted that two basic loss-of-function substitutions (R33Q and R42P) are close to acidic patches in the proposed PINK1–parkin interface, indicating that ionic interactions at this site may be important for efficient parkin phosphorylation. Increased auto-ubiquitination with unique ubiquitin chain patterns was observed for two other Ubl domain substitutions (G12R and T55I), suggesting that these substitutions, along with phosphorylation, increase parkin degradation. Moreover, Ubl domain phosphorylation decreased its affinity for the potential effector protein ataxin-3, which edits ubiquitin chain building by parkin. Overall, our work provides a framework for the mechanisms of parkin's loss-of-function, indicating an interplay between ARJP-associated substitutions and phosphorylation of its Ubl domain.

## Introduction

Mutations in the gene encoding parkin, an RBR RING1–in-between RING–Rcat (RING2) E3 ubiquitin ligase, are the most prevalent cause of autosomal recessive juvenile parkinsonism (ARJP)^3^ cases (1). In neurons, parkin is a key mediator of mitochondrial clearance via mitophagy following oxidative stress. Pathogenic mutations have been shown to impair this autophagic process (2, 3). Induction of mitophagy depends on the ubiquitin ligase activity of parkin, and deficiencies in ubiquitination attenuate this process, resulting in accumulation of oxidative damage. Such damage appears to be especially detrimental in neuronal and cardiac cells, as both cell types are characterized by high mitochondrial demands (4, 5).

The E3 ligase parkin consists of five distinct domains. The C-terminal region, common to all RBR E3 ligases, consists of a RING1 domain, required for E2-conjugating enzyme recruitment, a required-for-catalysis (Rcat, also termed RING2) domain that houses a catalytic cysteine needed for ubiquitin transfer, and an in-between RING domain that is structurally similar to the Rcat domain but lacks the catalytic site (6). Parkin also contains an accessory RING0 domain that occludes part of the Rcat domain (7) and an N-terminal auto-inhibitory ubiquitin-like (Ubl) domain that is proposed to be an important protein recruitment module during the ubiquitination cascade (8, 9). More than 100 pathogenic mutations are found throughout the entire parkin gene that predominantly result in missense substitutions in the N-terminal Ubl and the C-terminal Rcat domains (10).

Parkin exists in an auto-inhibited state, primarily through association of the Ubl domain with the remainder of the protein, that must be relieved to activate its ubiquitination function (11). Three-dimensional structures of parkin show that blockage of the E2 binding region by the Ubl domain causes auto-inhibition by preventing recruitment of the E2∼Ub conjugate and subsequent ubiquitin transfer (12–14). Recently, it has become clear that phosphorylation by the kinase PINK1 of both ubiquitin and the parkin Ubl domain significantly increases E3 ligase activity and subsequent induction of mitophagy (15–18). The three-dimensional structure of the phosphorylated Ubl (pUbl) domain shows that phosphorylation destabilizes this domain and causes a conformational change that weakens its auto-inhibitory interaction with the RING1 domain in parkin (19, 20). Conversely, phosphorylation of ubiquitin (pUb) at the equivalent serine 65 residue allosterically increases parkin's catalytic potential through binding to a site remote from both the Ubl domain binding region and the catalytic Rcat domain of the RBR (13, 14, 21–24). This complex regulatory mechanism, which requires phosphorylation of both ubiquitin and the parkin Ubl domain, remodels the tertiary structure of parkin by displacing the auto-inhibitory Ubl domain, thus allowing efficient recruitment of the E2∼Ub–conjugating complex.

In addition to acting as an auto-inhibitory module, it is less clear what the downstream roles of the Ubl domain are, especially in its phosphorylated state. The Ubl domain in its phosphorylated state is required for full E3 ligase activation (14, 24). However, how phosphorylation of the Ubl domain alters recruitment and ubiquitination (16, 18) of substrates is less well studied. One interesting class of parkin-interacting proteins or effectors are those containing ubiquitin-interacting motifs (UIMs), including ataxin-3, the PSMD4 (S5a) proteasomal subunit, and Eps-15 (9, 25). For example, the interaction of the parkin Ubl domain with the UIM regions of the deubiquitinating protein ataxin-3 is required for the unique ubiquitin-editing role of ataxin-3 (26, 27). In addition, parkin has been shown to ubiquitinate the PSMD4 subunit (28), and deletion of the Ubl domain decreases recruitment to the proteasome (29). Further, phosphorylation of the Ubl domain appears to have a role in the selectivity of ubiquitination sites in substrates such as Miro-1 (30).

Multiple potential outcomes of parkin ARJP mutations may lead to loss of function (23, 31). Substitutions in the Ubl domain (R42P and V56E) (32–34), RING1 domain (C212Y, R256C, C289G, and R275W) (35–37), and Rcat (RING2) domain (C41R and C441R) cause misfolding, resulting in either protein aggregates or proteasomal degradation. In contrast, other substitutions, including several in the Ubl (K27N, P37L, and R33Q) (38), RING1 (K211N and T240R) (36), and Rcat (RING2) domains (T415N and G430D) retain the native fold (7, 38–40) but suffer functional changes, such as lack of translocation to the mitochondria or an inability to perform the catalytic transthiolation reaction (41). It is also possible that mutations lead to inappropriate E3 ligase activation and subsequent auto-ubiquitination of parkin, which lead to its degradation. The effects of pathogenic mutations is further complicated when one considers that PINK1-mediated activation of parkin might be altered either through decreased efficiency of phosphorylation or modulation of ubiquitination by phospho-parkin.

Here we examine how pathogenic mutations in the parkin Ubl domain alter its ability to be phosphorylated by PINK1. We show that phosphorylation of the Ubl domain results in decreased stability of all variants harboring early-onset Parkinson's disease substitutions. Notably, in some cases, these lead to an increase in intrinsic auto-ubiquitination of parkin, a potential signal for degradation. In others, phosphorylation of the Ubl domain is dampened. Together, this work provides important mechanistic insights into the interplay between pathogenic mutations and phosphorylation of the parkin Ubl domain.

## Results

**Figure 1.**
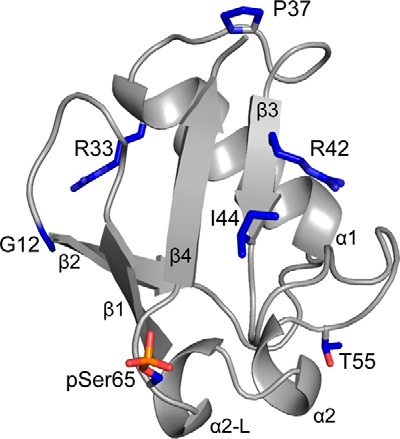
**Schematic of the parkin Ser-65–phosphorylated Ubl domain.** The secondary structure and the locations of substituted residues (*blue*) are shown. The position of phosphoserine 65 is also highlighted (*red*). This figure was produced using PDB coordinates from 5TR5.

The Ubl domain in parkin adopts a classical β-grasp fold comprising four β-strands, a longer α-helix that runs across one side of the β-sheet and a shorter α-helix that precedes Ser-65, the site of PINK1 phosphorylation (Fig. 1). In the Ubl domain and ubiquitin, the side-chain hydroxyl group of Ser-65 is hydrogen-bonded to the backbone amide of Asp-62 to stabilize this loop. The three-dimensional structure of the pSer-65 Ubl (pUbl) domain (20) shows that phosphorylation causes loss of the Ser-65 hydrogen bond, formation of a short helix-like structure, and rotation of the loop to expose the phosphate group to solvent (Fig. 1). A similar structural change that exposes phospho-Ser-65 has recently been shown for phospho-ubiquitin (42). Approximately 20 ARJP missense substitutions have been identified in the parkin Ubl domain that contribute to early-onset forms of Parkinson's disease (PD). Most of these substitutions are distributed uniformly throughout the Ubl domain structure (Fig. 1). To assess how these mutations alter the overall function of Ser-65-phosphorylated parkin, a subset of substitutions in the Ubl domain, including G12R (Ubl^G12R^), P37L (Ubl^P37L^), R42P (Ubl^R42P^), R33Q (Ubl^R33Q^), and T55I (Ubl^T55I^), was examined for efficiency of phosphorylation by PINK1, effects on protein stability, alterations in ubiquitination activity, and interaction strength with a proposed partner protein, ataxin-3.

### ARJP mutants lead to altered phosphorylation efficiencies

**Figure 2.**
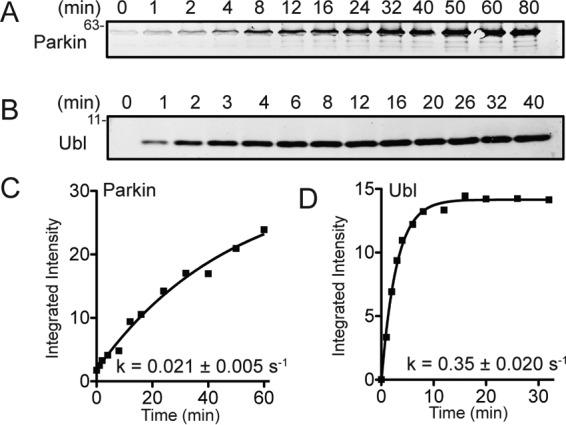
**PINK1-mediated phosphorylation of full-length parkin and Ubl domain monitored by Western blotting.** *A–D*, each reaction monitored 1 μm parkin (*A* and *C*) or Ubl domain (*B* and *D*) using either 50 nm (Ubl) or 500 nm (parkin) PINK1 for the specified times. Detection utilized a pSer-65–specific antibody with DyLight680-conjugated secondary antibody. Fluorescence was imaged and quantified at 700 nm and plotted to determine relative rate constants (k_obs_) of phosphorylation.

To identify how PD-causing mutations might alter the ability of parkin to be phosphorylated and, therefore, activated, the efficiency to phosphorylate full-length parkin compared with the isolated Ubl domain by *Pediculus humanus* PINK1 was examined. Although Phos-Tag SDS-PAGE can be used to monitor this conversion, we found that this resulted in poor resolution and “laddering” of bands that made quantifying the amounts of each protein difficult, especially for full-length parkin. Instead, we used a pSer-65 parkin-specific antibody and measured fluorescence at 680 nm from the DyLight fluorophore attached to a secondary antibody as a measure of phosphoprotein concentration. This approach was highly reproducible for both the pUbl domain and Ser-65–phosphorylated full-length parkin (pParkin) and produced only negligible background from unphosphorylated versions of these proteins, even at short reaction times (30 s). Time course phosphorylation reactions showed that both proteins could be phosphorylated, but with more than an entire order of magnitude difference in efficiency. Strikingly, the isolated Ubl domain was phosphorylated ∼16-fold faster than the full-length protein, even when 10-fold more PINK1 was used to phosphorylate the full-length parkin (Fig. 2). The poorer phosphorylation rate for full-length parkin is consistent with three-dimensional structures of parkin (13, 14) and chemical shift perturbation experiments by NMR spectroscopy (14, 20). These experiments show that the tether region following the in-between RING domain protects the parkin Ser-65 phosphorylation site in the auto-inhibitory state of the E3 ligase.

**Figure 3.**
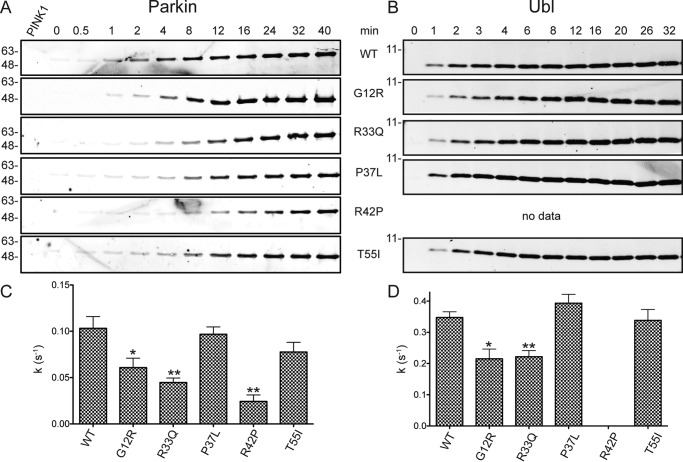
*A* and *B*, time course phosphorylation assays for full-length parkin (*A*) and the Ubl domain (*B*) carrying early-onset Parkinson's disease substitutions. Relative rates of phosphorylation were determined as shown in Fig. 2 and as described under “Experimental procedures.” *C* and *D*, rates were calculated from global fits of three independent experiments ± S.E. (represented by *error bars*) and plotted for parkin (*C*) and the Ubl domain (*D*). Statistically significant deviations for each substituted protein with respect to WT protein are indicated (*, *p* ≤ 0.05; **, *p* ≤ 0.01), as determined by a two-tailed *t* test. The Ubl^R42P^ protein was unfolded and insoluble and could not be examined.

The relative rates of Ser-65 phosphorylation were examined using time course experiments for full-length parkin and isolated Ubl domain variants carrying PD state substitutions (G12R, R33Q, P37L, R42P, and T55I) (Fig. 3). These experiments showed that the P37L and T55I substitutions were phosphorylated at rates comparable with the WT proteins for both isolated Ubl domain and full-length proteins. In contrast, the G12R and R33Q substitutions reproducibly resulted in 30–50% impaired phosphorylation by PINK1 relative to the WT proteins. The poorest phosphorylation substrate was the R42P substitution in full-length parkin. Phosphorylation of the isolated Ubl^R42P^ domain could not be examined because this substitution unfolds this domain *in vitro* (34). In addition, the Ubl^I44A^ domain was phosphorylated about 20-fold poorer than the WT Ubl domain (data not shown), consistent with previous observations for ubiquitin (43). These experiments show that substitutions at Gly-12, Arg-33, and Arg-42 are detrimental to parkin phosphorylation by PINK1.

### Phosphorylation of the parkin Ubl domain uniformly compromises its stability

**Figure 4.**
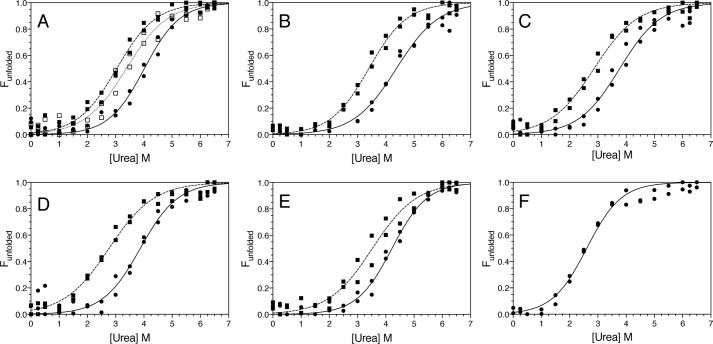
**Phosphorylation at Ser-65 uniformly decreases the stability of the parkin Ubl domain carrying PD substitutions.** *A–F*, denaturation curves derived from urea-mediated unfolding experiments measured at 218 nm by CD spectropolarimetry. Shown are data for Ubl (●), pUbl (■), and Ubl^S65E^ (□) (*A*); Ubl^G12R^ (●) and pUbl^G12R^ (■) (*B*); Ubl^R33Q^ (●) and pUbl^R33Q^ (■) (*C*); Ubl^P37L^ (●) and pUbl^P37L^ (■) (*D*); Ubl^T55I^ (●) and pUbl^T55I^ (■) (*E*); and Ubl^I44A^ (●) (*F*). All data were fit as described under “Experimental procedures.”

Some ARJP substitutions in parkin lead to misfolding of the protein, which can lead to intracellular aggregation and increased turnover (35–37). This has not been examined in the context of the phosphorylated state. To address this, we used chemical and thermal denaturation methods monitored by CD spectropolarimetry to compare the stabilities of ARJP-substituted Ubl domain proteins in the presence and absence of Ser-65 phosphorylation (Fig. 4, Fig. S1, Table 1). The CD spectra recorded at 5 °C showed that both unphosphorylated and phosphorylated proteins had minima near 205 nm and 222 nm, consistent with the α/β nature of the Ubl domain. In the absence of phosphorylation, the urea-mediated unfolding profiles had smooth sigmoidal transitions, consistent with a two-state unfolding process. These experiments showed that all substitutions (Ubl^G12R^, Ubl^R33Q^, Ubl^P37L^, and Ubl^T55I^) resulted in small (0.4–1.1 kJ/mol) changes in the overall structural stability of the Ubl domain. These differences were much less detrimental than other Parkinson's substitutions (R42P, A46P, and V56E) that cause misfolding of the isolated Ubl domain (38). For comparison, we also examined the stability of the Ubl domain carrying an I44A substitution (Ubl^I44A^). This substitution is frequently used with Ubl proteins because of its central role in protein interactions (11, 44). Unexpectedly, this protein had the poorest stability of all proteins studied. Further, ^1^H-^15^N NMR spectra of the Ubl^I44A^ domain at room temperature showed several large chemical shift changes indicative of a possible structural change near the Ile-44 site (Fig. S2).

**Table 1:** **Energetics of unfolding for Ubl and pUbl domain proteins**

Protein	Unfolding energies			Effect of phosphorylation	
	D_50%_	*m*	Δ*G*_*u*_^H2O^	ΔΔ*G*_*u*_^D50%^	ΔΔ*G_u_*
	*(m)*	*(kJ mol*^−*1*^ *m*^−*1*^*)*	*(kJ mol*^−1^*)*	*(kJ mol*^−1^*)**^a^*	*(kJ mol*^−*1*^*)**^b^*
Ubl	4.10 ± 0.04	3.7 ± 0.2	14.7 ± 1.0	−*^c^*	
pUbl	3.12 ± 0.05	3.3 ± 0.2	10.1 ± 0.6	3.4 ± 0.2	1.5
S65E	3.38 ± 0.06	3.1 ± 0.2	10.8 ± 0.8	2.4 ± 0.3	
P37L	3.87 ± 0.07	3.5 ± 0.3	13.5 ± 1.2		
pP37L	2.77 ± 0.05	2.9 ± 0.2	8.0 ± 0.5	3.5 ± 0.3	1.7
G12R	4.37 ± 0.05	3.1 ± 0.2	13.4 ± 0.9		
pG12R	3.40 ± 0.04	3.4 ± 0.2	11.6 ± 0.6	3.2 ± 0.2	2.0
R33Q	3.77 ± 0.06	3.2 ± 0.2	12.0 ± 0.9		
pR33Q	2.86 ± 0.05	3.0 ± 0.2	8.6 ± 0.5	2.8 ± 0.2	1.8
T55I	4.22 ± 0.04	3.7 ± 0.2	15.7 ± 0.9		
pT55I	3.52 ± 0.06	3.0 ± 0.2	10.4 ± 0.8	2.3 ± 0.2	1.9
I44A	2.61 ± 0.04	3.8 ± 0.3	9.9 ± 0.7		
pI44A	−*^c^*	−*^c^*	−*^c^*	−*^c^*	1.1

*^a^* Difference in stability between unphosphorylated and phosphorylated Ubl proteins measured by chemical denaturation methods. Calculated from Equation 5 under “Experimental procedures” using Δ*D*_50%_ = *D*_50%_(Ubl) − *D*_50%_(pUbl) or similar for substituted proteins.

*^b^* Difference in stability between unphosphorylated and phosphorylated Ubl proteins measured by thermal unfolding methods. Calculated using Equation 6 under the “Experimental procedures” using Δ*T_m_* = *T_m_* (Ubl) − *T_m_* (pUbl).

*^c^* −, not measured.

Upon Ser-65 phosphorylation, all Ubl domain proteins (pUbl) had unfolding midpoints that were ∼1.0 m urea lower than their unphosphorylated versions, translating into about 3 kJ/mol decreased stability upon phosphorylation. Similarly, thermal unfolding experiments showed that all substituted pUbl proteins were about 1.5–2 kJ/mol lower stability in their phosphorylated states (Fig. S1). The structure of the pUbl domain (24) shows that phosphorylation results in loss of the hydrogen bond between the Ser-65 side chain and the Asp-62 backbone amide and the subsequent exposure of Ser-65. The similar decrease in stabilities for the phosphorylated Ubl domain proteins indicates that this structural change consistently occurs in all pUbl variants regardless of the ARJP substitution.

### Alterations in ubiquitination by a phosphorylated Ubl domain

**Figure 5.**
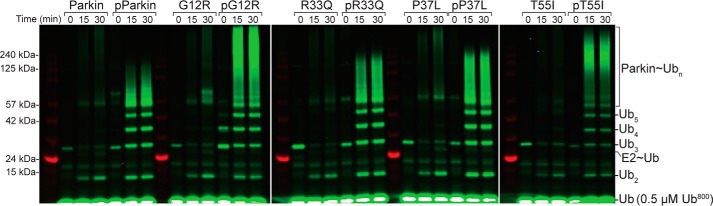
**Substitutions in the Ubl domain alter parkin autoubiquitination.** The assay shows ubiquitination products monitored using fluorescently labeled Ub (*Ub^800^*). Each ubiquitination series shows parkin and phosphoparkin taken at the 0, 15, and 30-min time points for the WT and four different substitutions. All other ubiquitination conditions are described under “Experimental procedures.”

Phosphorylation of the Ubl domain is required for parkin to reach its full E3 ligase activity. To determine how PD substitutions alter the ubiquitination efficiencies upon phosphorylation, we examined the ubiquitination profiles of phosphorylated parkin carrying PD substitutions (Fig. 5). All assays included pUb and were assessed by following the formation of free polyubiquitin chains and auto-ubiquitinated parkin using a fluorescently labeled Ub probe. When comparing nonphosphorylated parkin species, substitutions at G12R and P37L had minor ubiquitination increases in activity compared with the WT E3 ligase, whereas R33Q and T55I were more similar. As expected, Ser-65-phosphorylation of parkin in the presence of pUb greatly increased ubiquitination for all proteins examined, consistent with current models that these two events are needed for full ubiquitin activation of parkin (15–18). However, unexpectedly, some Parkinson's substitutions in the Ubl domain resulted in ubiquitination profiles suggestive of increased ubiquitination activity compared with the WT protein. This was especially apparent for pUbl^G12R^- and pUbl^T55I^-substituted proteins. Even more striking were the differences in the patterns of auto-ubiquitination between the phosphorylated variants. For example, the pUbl^G12R^ and pUbl^T55I^ parkin proteins reproducibly formed the bulk of polyubiquitin chains at higher molecular weights (>125 kDa) compared with the WT protein. In contrast, the pUbl^R33Q^ and pUbl^P37L^ proteins had ubiquitination efficiencies and formed chains with a more finite length (<125 kDa), similar to WT pParkin. These results indicate that phosphorylation of parkin, in combination with some PD substitutions, alters the ubiquitination efficiency and ubiquitin chain pattern.

### Phosphorylation modulates interactions with ataxin-3

The parkin Ubl domain may function as a recruitment module for accessory proteins, substrates, or a docking module for ubiquitinated proteins (8, 9). Further, large-scale proteomics screens have shown that phosphorylation of parkin can have a significant impact on the interactions of parkin with other proteins (45). Because mutation and phosphorylation of the Ubl domain had effects on both stability and ubiquitination, we hypothesized that these might act synergistically to modulate interactions with a potential effector protein. One particularly well-studied group of parkin-interacting proteins are those containing UIM sequences known to shuttle ubiquitin and ubiquitin-like proteins (46). Parkin has been shown to recruit at least three UIM proteins, including ataxin-3, the PSMD4 subunit of the 19S proteasome, and Eps15 (9, 25, 47, 48). In particular, the deubiquitinating enzyme ataxin-3, which includes three tandem UIMs, can be recruited by the Ubl domain from parkin (47) and is necessary to edit ubiquitin chain building by parkin (26, 27). We used the entire UIM region (residues 194–361, ataxin^194–361^) as a surrogate Ubl-interacting protein to examine how phosphorylation of the parkin Ubl domain affects its interaction with ataxin-3 and how PD mutations might further modulate this interaction.

**Figure 6.**
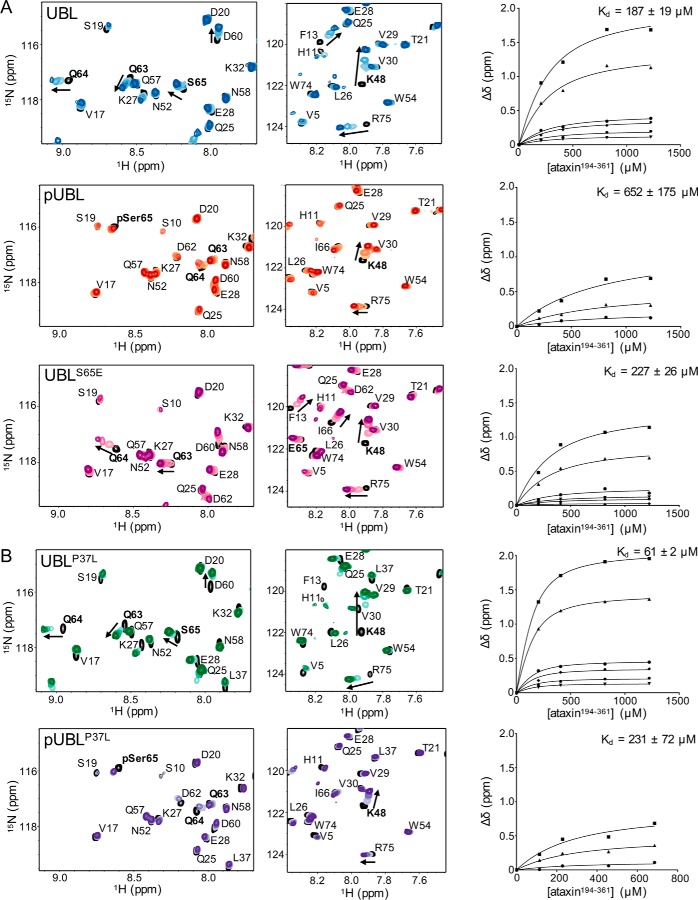
**ARJP substitutions and phosphorylation of Ubl alter the affinity with ataxin-3.** A series of ^1^H-^15^N HSQC spectra were used to monitor the interactions between the ^15^N-labeled Ubl variants and the unlabeled tandem UIM region from ataxin-3 (ataxin^194–361^). Regions of the spectra for Ubl (*A*, *top*), pUbl (*center*), and Ubl^S65E^ (*bottom*) and Ubl^P37L^ and pUbl^P37L^ (*B*) are shown with common residues that shift upon ataxin^194–361^ addition (*colored contours*) compared with the Ubl domain alone (*black contours*). Residues used for affinity calculations are indicated by *arrows* and are plotted as change in chemical shift for either ^1^H or ^15^N for Ile-44 (●), Lys-48 (■), Glu-49 (▴), Gln-63 (♦), Gln-64 (▾), and Ser-65/pSer-65/S65E (●). The curves were obtained from a global fit for a particular dataset to obtain a best fit value for *K_d_*. Titration experiments used 150 μm Ubl domain (75 μm pUBL^P37L^) in 25 mm HEPES buffer and 100 mm NaCl (pH 7.0) at 25 °C.

The C-terminal region of ataxin-3 containing all three UIM sequences (ataxin^194–361^) was titrated into ^15^N-labeled Ubl or pUbl domain proteins, and the interactions were monitored by chemical shift changes in the resulting ^1^H-^15^N NMR spectra. Titration of ataxin^194–361^ into the WT Ubl domain showed the largest changes in signals, corresponding to Ile-44, Lys-48, and Glu-49 near the Ile-44 hydrophobic patch; Gln-63, Gln-64, and Ser-65 in the Ser-65 phosphorylation site; and Arg-75 at the C terminus (Fig. 6*A*, *top panels*). Fitting of these data resulted in an apparent dissociation constant (*K_d_*) between ataxin^194–361^ and the parkin Ubl domain of *K_d_^Ubl^* = 187 ± 19 μm, similar to that reported previously (47).

An identical titration of ataxin^194–361^ into the [^15^N]pUbl domain showed that the interaction between these proteins weakened by more than 3-fold upon phosphorylation (*K_d_^pUbl^* = 652 ± 175 μm). Notably, this was accompanied by negligible chemical shift changes in Gln-63, Gln-64, and pSer-65 and smaller changes to residues such as Lys-48 (Fig. 6*A*, *center panels*). Interestingly, the phosphomimetic Ubl^S65E^ protein (Fig. 6*A*, *bottom panels*) had an affinity (*K*_*d*_^*S*65*E*^ = 227 ± 26 μm) and chemical shift changes akin to the WT protein, perhaps highlighting a weaker charge effect than phosphorylation at the Ser-65 site.

The interactions of ataxin-3 with the Ubl^P37L^ and pUbl^P37L^ domains were also examined to identify how a Parkinson's disease–substituted parkin combined with phosphorylation might further alter this interaction (Fig. 6*B*). The Ubl^P37L^ domain variant was chosen because its stability change upon phosphorylation was similar to the WT protein and it could be phosphorylated in large enough quantities for NMR experiments. NMR titration experiments revealed that the Ubl^P37L^ domain bound nearly 3-fold tighter to ataxin^194–361^ (*K*_*d*_^*P*37*L*^ = 61 ± 2 μm) than the WT protein. However, upon phosphorylation, the affinity for ataxin^194–361^ decreased about 4-fold to a value more similar to that for the WT protein lacking phosphorylation (*K*_*d*_^*pP*37*L*^ = 231 ± 72 μm). Similar to the pUbl domain, the pUbl^P37L^ domain experienced little change in the positions of Gln-63, Gln-64, and pSer-65 compared with the unphosphorylated form in the presence of ataxin^194–361^. Overall, these data indicate that phosphorylation dampens the Ubl domain interaction with the UIM-containing protein ataxin-3 through loss of interactions with residues near the Ser-65 site.

## Discussion

Early-onset Parkinson's disease substitutions are found throughout the E3 ligase parkin but appear to be concentrated in two domains: the N-terminal Ubl domain and the C-terminal Rcat (RING2) domain (1, 10, 49). Some of these substitutions (K27N, R42P, and A46P) cause decreased stability or unfolding of the isolated Ubl domain (38) but appear to be more stable in the complete protein (50). In the full-length protein, substitutions, including K27N, R33Q, R42P, A46P, and V56E, have been shown to render parkin more susceptible to degradation (11, 32). Changes in levels of auto-ubiquitination or ubiquitin charging of parkin have been noted for PD-substituted proteins in the presence or absence of mitochondrial depolarization conditions (11, 36, 50). All of these observations paint a complex picture by which parkin regulation may be affected by both PD mutations and phosphorylation.

**Figure 7.**
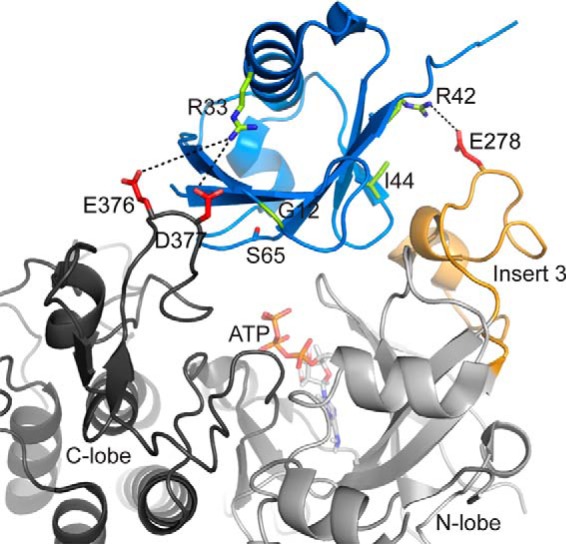
**Model of the parkin Ubl interaction with PINK1.** The model was constructed using the coordinates for PINK1 (PDB code 6EQI) and superposition of the Ubl domain structure with the bound ubiquitin in the structure. The ATP molecule from the cAMP-dependent protein kinase (PDB code 1ATP) was added as described previously (31). The model shows the activation loop of the C lobe (*gray*) and Insert3 of the N lobe of PINK1 against the Ubl domain (*blue*) and ATP (*orange*). The side chains of Glu-278, Glu-376, and Asp-377 were modeled in, as these were not visible in the crystal structure. PD substitutions for R33Q, R42P, and G12R are shown with potential ionic contacts to PINK1.

The Ubl domain must be phosphorylated by PINK1, typically under cellular oxidative stress conditions, to reach its full E3 ligase activity. In this work, three ARJP substitutions (G12R, R33Q, and R42P) showed marked decreases in phosphorylation efficiency, indicating that these substitutions will lead to overall lower E3 ligase activity. To provide a rationale for the decreased phosphorylation efficiency, we modeled the Ubl domain in place of ubiquitin in the recent structure of the PINK1–Ub complex (51). This model (Fig. 7) shows that the side chain of Arg-33 is located near Asp-376 and Glu-377 on the activation loop, whereas Arg-42 lies near Glu-278 in Insert3 of PINK1. These basic residues lie on opposite sides of the Ubl domain, contacting nearby acidic residues within the active-site bowl in PINK1. Other PD mutations, including R42H and R42C in the Ubl domain, presumably cannot make the appropriate ionic contacts with the PINK1 acidic residue (Glu-278). It is interesting that neither Arg-33 in the Ubl domain nor Asp-376/Glu-377 in PINK1 are particularly well conserved, providing a rationale for the significant differences between phosphorylation efficiencies of human parkin using different PINK1 species (52). As shown in previous work, the impaired phosphorylation efficiency of the Ubl^I44A^ domain results from its juxtaposition to Insert3 where it contacts Phe-196–Tyr-198 (51). The decreased phosphorylation of G12R is more difficult to reconcile. Nevertheless, this residue faces the bottom of the bowl, and it is conceivable that replacement with a larger residue (Ubl^G12R^) might create steric hindrance with the ATP in the active site. Thus, the decreased phosphorylation efficiencies of the Ubl domain carrying ARJP substitutions appear to be a direct reflection of improper recognition by PINK1 that would limit the ATP phosphoryl transfer step and provides a unifying mechanism underlying decreased overall E3 ligase activity.

After Ser-65 phosphorylation in PD-substituted parkin has occurred, protein stability may be altered, causing folding defects and subsequent degradation by the 26S proteasome. Phosphorylation of the Ubl domain results in consistent, uniform destabilization of this domain by ∼1–3 kJ/mol. This observation is consistent with the recent three-dimensional structure of pUbl (20) that shows that Ser-65 phosphorylation causes the loss of a hydrogen bond from the Ser-65 hydroxyl to the backbone carbonyl of Asp-62 and forces the phosphate group to an exposed position. We hypothesize that Ser-65 phosphorylation induces this conformational change in all ARJP proteins studied here rather that resulting in any dramatic unfolding. There may, however, be subtle differences in the full-length phosphorylated protein. For example, the isolated Ubl^R42P^ domain is completely unfolded (34). However, in the context of full-length parkin, this substitution does not result in degradation, likely because of protection by the RING1 domain where the Ubl domain packs. Interestingly, when the Ubl^R42P^ domain is fused to a carrier protein, it is efficiently packaged to the endoplasmic reticulum for degradation (53). Because phosphorylation of the Ubl in conjunction with pUb activation releases the pUbl domain from its RING1 binding site, it is possible that phosphorylation of cytosolic Parkin^R42P^ and other poorly folded Ubl domain proteins (A46P and V56E) promotes their degradation via endoplasmic reticulum-associated protein degradation (53) only when these phosphorylation steps are complete.

Parkinson's substitutions within the Ubl domain can lead to increased ubiquitination activity even in the absence of phosphorylation of the Ubl domain or pUb activation. For example, increased auto-ubiquitination has been observed previously for the R33Q, K27N, and R42P substitutions (11, 14). In this work, the pParkin^G12R^ and pParkin^T55I^ proteins both yielded increased ubiquitination and trended toward higher average molecular weight ubiquitination species of >125 kDa. Parkin has been shown to build multiple lysine chain types, including Lys-48–linked chains that would signal its degradation (17, 45). The increased auto-ubiquitination activity for pParkin^G12R^ and pParkin^T55I^ could signal premature degradation of these species through the 26S proteasomal pathway compared with WT parkin. For Parkin^G12R^, this may be less prominent because this protein exhibited an ∼40% slower phosphorylation rate, which would ultimately limit its ubiquitination activity.

The Ubl domain is an important module for protein interactions with the UIM-containing proteins ataxin-3 and Eps15 and the S5a subunit of the 19S proteasome (9, 25, 47, 48). In addition, the parkin Ubl domain forms a complex with the SH3 domain from endophilin-A1 that is required for parkin-mediated ubiquitination and has implications for some synaptic transmission defects observed in Parkinson's disease (54). Further, it has been shown that phosphorylation at Ser-65 is necessary (55) and increases the selectivity for the Lys-572 ubiquitination site in at least one substrate, Miro1, and leads to more efficient ubiquitination of a substrate overall (30). Deletion of the Ubl domain results in loss of ubiquitination of two known mitochondrial substrates, mitofusin 1/2 (56, 57) and Miro1 (30), but increased auto-ubiquitination, suggesting that the Ubl domain directs ubiquitination toward the substrate. Additionally, constitutively active parkin, achieved through an N-terminal His_6_ tag, does not ubiquitinate Miro1 (30), indicating that simple dissociation of the Ubl domain is not sufficient for activity. Therefore, phosphorylation of the Ubl domain may provide a control mechanism for protein recruitment and ubiquitination site specificity.

Surprisingly, phosphorylation of the Ubl domain leads to an approximate 3-fold decrease in affinity for a potential binding partner, ataxin-3. This would suggest that phosphorylation of the Ubl domain modulates the ubiquitin-editing function of ataxin-3 through weakening the affinity for the UIM regions known to be needed for ataxin's deubiquitinating activity with parkin (26, 27). On the other hand, the ARJP substitution Ubl^P37L^ showed increased affinity for ataxin-3, which was reversed through Ubl phosphorylation. This would suggest that this substitution might increase the deubiquitinating activity of ataxin-3 through a stronger interaction, giving rise to premature/shortened ubiquitin chains. In addition, the Ser-65 region in the Ubl domain appears to be central for the ataxin-3 interaction, given the perturbations observed in NMR titration experiments that are nullified when the Ubl domain is phosphorylated. Biologically, phosphorylation at Ser-65 may inhibit the interaction between parkin and ataxin-3, allowing activated parkin to proceed with translocation to the mitochondria and substrate ubiquitination. This observation may be very specific for the UIM-containing proteins because the Ser-65 region in the Ubl domain does not appear to be important for recruitment of non-UIM protein such as endophilin-A1 based on limited chemical shift changes in this region and remoteness of the Ser-65 region from the endophilin interface (54).

Our work shows that the combination of phosphorylation and PD mutations within the parkin Ubl domain compromise function through multiple pathways. In one pathway, substitutions such as G12R, R33Q, and R42P diminish the ability of parkin to be phosphorylated, resulting in poorer availability of fully activated E3 ligase. Alternatively, substitutions such as T55I lead to abnormally high amounts of auto-ubiquitination, which could signal premature degradation of parkin. Finally, substitutions such as P37L are more complex because they have no noticeable difference in phosphorylation efficiency or ubiquitination activity. In these instances, it is plausible that parkin loss of function may be due to altered recognition of one or more of the many parkin substrates at the mitochondrial membrane or beyond. Together, these results demonstrate that the loss-of-function phenotype arising from PD substitutions in the Ubl domain results from a multitude of complex pathways that uniformly hinder the mitophagic process thought to protect against neurodegeneration.

## Experimental procedures

### Protein constructs and purification

All human parkin (1–465) and Ubl domain (1–76) constructs were encoded as His–small ubiquitin–like modifier fusion proteins as described previously (11). Disease state mutations were introduced using a modified site-directed mutagenesis protocol (58). Proteins were expressed in *Escherichia coli* BL21(DE3) cells in Luria broth or M9 minimal medium (59). For purification of expressed proteins, cells were harvested, resuspended in lysis buffer (50 mm Tris, 500 mm NaCl, 0.5 mm TCEP, and 25 mm imidazole (pH 8.0)) and lysed using an EmulsiFlex-C5 homogenizer (Avestin). All proteins were purified by Ni^2+^-NTA affinity chromatography using a HisTrap FF column on an AKTA FPLC (GE Healthcare) and cleaved with Ulp1 protease (∼1:50 ratio protease:protein). Following cleavage, proteins were repassed over the HisTrap FF column, collecting the flow-through and applying this to a HiLoad Superdex75 gel filtration column pre-equilibrated in 25 mm HEPES, 100 mm NaCl, and 0.5 mm TCEP (pH 7.0). Proteins for phosphorylation experiments were aliquoted, flash-frozen, and stored at −80 °C until use. Proteins for ubiquitination experiments were used immediately after purification or phosphorylation.

The C-terminal portion of ataxin-3 (residues 194–361) containing three UIM regions was expressed as a His-tagged protein in *E. coli* BL21(DE3) cells in Luria broth medium. Cells were grown at 37 °C and induced with 0.63 mm isopropyl 1-thio-β-d-galactopyranoside when an *A*_600_ of 0.8 was reached. The temperature was then reduced to 16 °C, and cells were allowed to grow overnight. Cells were harvested and resuspended in a lysis buffer (20 mm NaH_2_PO_4_, 20 mm Na_2_HPO_4_, 300 mm NaCl, and 10 mm imidazole (pH 8.0)) and lysed using an EmulsiFlex-C5 homogenizer (Avestin). The protein was purified by Ni^2+^-NTA affinity as described above and eluted with 20 mm NaH_2_PO_4_, 20 mm Na_2_HPO_4_, 300 mm NaCl, and 250 mm imidazole (pH 8.0). The His tag was cleaved using tobacco etch virus protease during overnight dialysis, and the untagged ataxin-3 protein was purified through reapplication to a Ni^2+^-NTA affinity column.

GST-tagged *P. humanus* PINK1 (residues 128–575) was expressed in *E. coli* BL21(DE3) cells in Luria broth medium. Cells were grown at 37 °C until an *A*_600_ of 0.8 was reached, at which point expression was induced with 0.5 mm isopropyl 1-thio-β-d-galactopyranoside, and the incubation temperature was lowered to 16 °C for 18 h. Expressed proteins were purified on a GSTrap FF column (GE Healthcare) in lysis buffer (50 mm Tris, 150 mm NaCl, 1 mm EDTA, and 1 mm DTT (pH 7.4)) and eluted with freshly prepared elution buffer (50 mm Tris, 150 mm NaCl, 1 mm DTT, and 10 mm GSH (pH 8.0)). Following elution, GSH was removed from PINK1 by two 2L dialysis changes against 50 mm Tris, 50 mm NaCl, and 3 mm DTT (pH 10), followed by a final 2L dialysis against 50 mm Tris, 50 mm NaCl, and 3 mm DTT (pH 7.5).

Ubiquitin with an N-terminal cysteine (dissolved in 25 mm Tris, 100 mm NaCl, and 0.5 mm EDTA (pH 7.4)) was expressed and purified as described previously. The purified protein was modified with 5 mm Dylite800 (dissolved in 100% isopropanol) for 1 h and quenched with 5 mm DTT. Excess Dylite800 label was removed using a HiLoad Superdex75 gel filtration column. Aliquots of Dylite800-labeled ubiquitin (Ub^800^) were flash-frozen and stored at −80 °C until needed.

### Phosphorylation of parkin and Ubl domain

For phosphorylation time course experiments, purified GST-PINK1 was used to phosphorylate all parkin and Ubl domain variants. Proteins (1 μm final concentration) were diluted in 50 mm Tris, 50 mm NaCl, and 3 mm DTT (pH 7.5) to obtain a PINK1:parkin ratio of 1:2 (parkin) or 1:20 (Ubl domain) in a total volume of 200 μl. Phosphorylation reactions were initiated by addition of 20 mm MgCl_2_ and 10 mm ATP (pH 7.5) and allowed to proceed at 25 °C. At specified time intervals, aliquots (10 μl) were quenched with 3× SDS loading buffer (containing 100 mm EDTA) and boiled at 95 °C for 5 min. Samples were then analyzed by SDS-PAGE, and unstained gels were processed using a shared nitrocellulose membrane (0.2-μm pore size) using a semidry iBlot2 apparatus (Invitrogen). Membranes were rinsed with water and blocked with 10× BSA (Pierce) in TBS for 1 h at room temperature. A final rinse with TBS + 10% aqueous Tween 20 (TBST) was used prior to incubation with primary antibody (anti-pSer-65 parkin, Ubiquigent, catalog no. 68-0056-100) overnight at 4 °C. Following this, membranes were rinsed with three to four washes of TBST, incubated with a fluorescent secondary antibody (anti-sheep Dylight 680 nm, Pierce, catalog no. SA5-10058) for 45 min, and washed again three times with TBST. Membranes were scanned by an Odyssey Imaging system (LiCor), and fluorescence intensity was measured at 700 nm.

Fluorescence intensity was plotted against time for each parkin variant, demonstrating the formation of phosphorylated parkin (pParkin). The formation of pParkin can be described chemically as a second-order rate equation. Because the concentration of ATP is present at a 10,000-fold excess and largely unchanged over the reaction, analysis of the data was simplified to a pseudo-first-order equation that yielded the rate of formation of pParkin as a function of time.

### Unfolding experiments

Purified protein samples were dialyzed against three changes of 20 mm KH_2_PO_4_ (pH 7.0) and 4 °C to remove chloride ions. Protein concentrations were determined by measuring the absorbance at 280 nm using a nanodrop instrument (DeNovix). A series of samples containing 0–6.5 M urea was prepared by diluting equivalent amounts of the desired protein into buffer and adding the appropriate amount of 10 m urea. Samples were incubated overnight at 4 °C. Chemical denaturation experiments were performed in duplicate using a JASCO J-810 CD spectropolarimeter equipped with a Peltier temperature control. Data points were collected as an average of five replicate scans using a 1-mm path length cuvette at 5 °C. The resulting CD ellipticity at 218 nm (*Y*_obs_) was plotted as a function of urea concentration [D] and fit based on a two-state unfolding transition between folded (F) and unfolded (U) forms of the protein (Equations 1–3). The free energy difference (Δ*G*_*u*_^H2O^) between folded and unfolded proteins and the slope of the unfolding transition (*m*) were determined as described previously (34, 38, 60) using nonlinear regression (GraphPad Prism) based on Equation 4. The baselines prior to (*Y_f_*) and following (*Y_u_*) the unfolding transition were accounted for in all fittings.

(1) $${\text{Ubl}}_{\text{folded}}\left(F\right)⇄{\text{Ubl}}_{\text{unfolded}}\left(U\right)$$

(2) $$K_{u}\text{=}\frac{{\text{Ubl}}_{\text{fold}}}{{\text{Ubl}}_{\text{unfold}}}=\frac{F}{U}$$

(3) $$K_{u}\text{=}\frac{\exp\left(\Delta G_{u}^{{\text{H}}_{2}\text{O}}+m\left[D\right]\right)}{-\text{RT}}$$

(4) $$Y_{\text{obs}}\text{=}\frac{Y_{f}+Y_{u}K_{u}}{1+K_{u}}$$

To compare the stabilities between nonphosphorylated and phosphorylated proteins, the midpoints (D_50%_) of each curve were used, and the difference in stability (ΔΔ*G*_*u*_^D50%^) was calculated from Equation 5, where *m̄* is the average slope for the two proteins.

(5) $$\Delta \Delta G_{u}^{{\text{D}}_{50\%}}=\bar{m}\Delta D_{50\%}$$

For thermal unfolding, the protein samples were prepared as above, and the experiments were performed in duplicate. The temperature was increased from 5–85 °C at 5 °C per minute, and unfolding was monitored at 206 nm. The data were analyzed as described previously (48) by assessing the difference in stability (ΔΔ*G_u_*) between nonphosphorylated and phosphorylated proteins based on Equation 6, where Δ*H*_(non)_ and *T_m_*_(non)_ are the enthalpy and unfolding midpoint for the nonphosphorylated protein, and Δ*T_m_* is the difference in unfolding temperature between nonphosphorylated and phosphorylated proteins (61).

(6) $$\Delta \Delta G_{u}=\Delta T_{m}\times \frac{\Delta H_{\left(\text{non}\right)}}{T_{m\left(non\right)}}$$

### Titration experiments to measure the Ubl and pUbl interaction with ataxin-3

A series of NMR samples was prepared by diluting the ^15^N-labeled Ubl domain with the appropriate amount of ataxin-3 and NMR buffer (25 mm HEPES, 100 mm NaCl, 0.5 mm TCEP (pH 7.0), and 10% (v/v) D_2_O). An internal reference of 200 μm 2,2-dimethyl-2-silapentanesulfonic acid was used, and 300 μm of imidazole was used as an internal pH indicator. The NMR data were collected using a Varian Inova 600-MHz NMR spectrometer. Data were processed using NMRPipe and analyzed using NMRViewJ. Dissociation constants (*K_d_*) were calculated by manually recording the ^1^H or ^15^N chemical shift for each residue after ataxin^194–361^ titration and subtracting the original chemical shift. These Δδ values were plotted against the total added [ataxin^194–361^], and all curves were globally fit for a single *K_d_* using GraphPad Prism with Equation 7,

(7) $$\Delta \delta =\frac{\left[\left(P_{t}+L+K_{d}\right)-{\left[{\left(P_{t}+L+K_{d}\right)}^{2}-\left(4\times L\times P_{t}\right)\right]}^{0.5}\right]}{2}$$

where *P_t_* is the Ubl domain concentration, *L* is the ataxin^194–361^ concentration, and Δδ is the calculated change in chemical shift.

### Parkin ubiquitination assays

Reactions were completed using 4 μm Ub, 0.5 μm pUb, 0.5 μm Ub^800^, 0.5 μm UbcH7, 0.1 μm Uba1, 5 mm MgATP, 50 mm HEPES (pH 7.5), and 1 μm WT or substituted parkin and pParkin. Reactions were quenched at 0, 15, and 30 min using 3× SDS sample buffer and 1 m TCEP. Gradient gels (4–12% BisTris Plus, Thermo Fisher Scientific) were used with MES running buffer (250 mm MES, 250 mm Tris, 0.5% SDS, and 5 mm EDTA (pH 7.3)). Gels were scanned by the Odyssey Imaging system (LiCor), and fluorescence intensity was measured at 700 nm and 800 nm.

## Author contributions

J. D. A., K. M. D., and G. S. S. conceptualization; J. D. A., K. M. D., R. L., and M. R. data curation; J. D. A., K. M. D., R. L., and G. S. S. formal analysis; J. D. A. and K. M. D. investigation; J. D. A., K. M. D., and R. L. methodology; J. D. A., K. M. D., and R. L. writing-original draft; J. D. A., K. M. D., and G. S. S. writing-review and editing; G. S. S. supervision; G. S. S. funding acquisition; G. S. S. validation.

## Supplementary Material

Supporting Information

## Footnotes

3 The abbreviations used are:

ARJP: autosomal recessive juvenile parkinsonism
Rcat: required-for catalysis
Ubl: ubiquitin-like
Ub: ubiquitin
UIM: ubiquitin-interacting motif
TCEP: tris(2-carboxyethyl)phosphine
Ni^2+^-NTA: nickel-nitrilotriacetic acid
GST: glutathione S-transferase
TBS: Tris-buffered saline
PD: Parkinson's disease
pUb: phosphorylated ubiquitin
pUbl: phosphorylated ubiquitin-like domain
RING: really interesting new gene.
