# Supplementary material for: Impact of altered phosphorylation on loss of function of juvenile Parkinsonism–associated genetic variants of the E3 ligase parkin
Source: J Biol Chem. 2018 Mar 12;293(17):6337–48. doi: 10.1074/jbc.RA117.000605 (PMC5925814; doi:10.1074/jbc.RA117.000605)
Supplement: Supporting Information [file supp_293_17_6337__index.html]

Impact of altered phosphorylation on loss of function of juvenile Parkinsonism-associated genetic variants of the E3 ligase parkin — Altered Phosphorylation in ARJP-variants of Parkin — Impact of altered phosphorylation on loss of function of juvenile Parkinsonism–associated genetic variants of the E3 ligase parkin — Altered phosphorylation in ARJP-variants of parkin — Supporting Information 

# Impact of altered phosphorylation on loss of function of juvenile Parkinsonism–associated genetic variants of the E3 ligase parkin

## Supporting Information

- Figure S1 - Supplemental figure and legend
- Figure S2 - Supplemental figure and legend
