# Supplementary material for: Impact of altered phosphorylation on loss of function of juvenile Parkinsonism–associated genetic variants of the E3 ligase parkin
Source: J Biol Chem. 2018 Mar 12;293(17):6337–48. doi: 10.1074/jbc.RA117.000605 (PMC5925814; doi:10.1074/jbc.RA117.000605)
Supplement: Supporting Information [file supp_RA117.000605_133409_2_supp_89542_p56rr6.pdf]

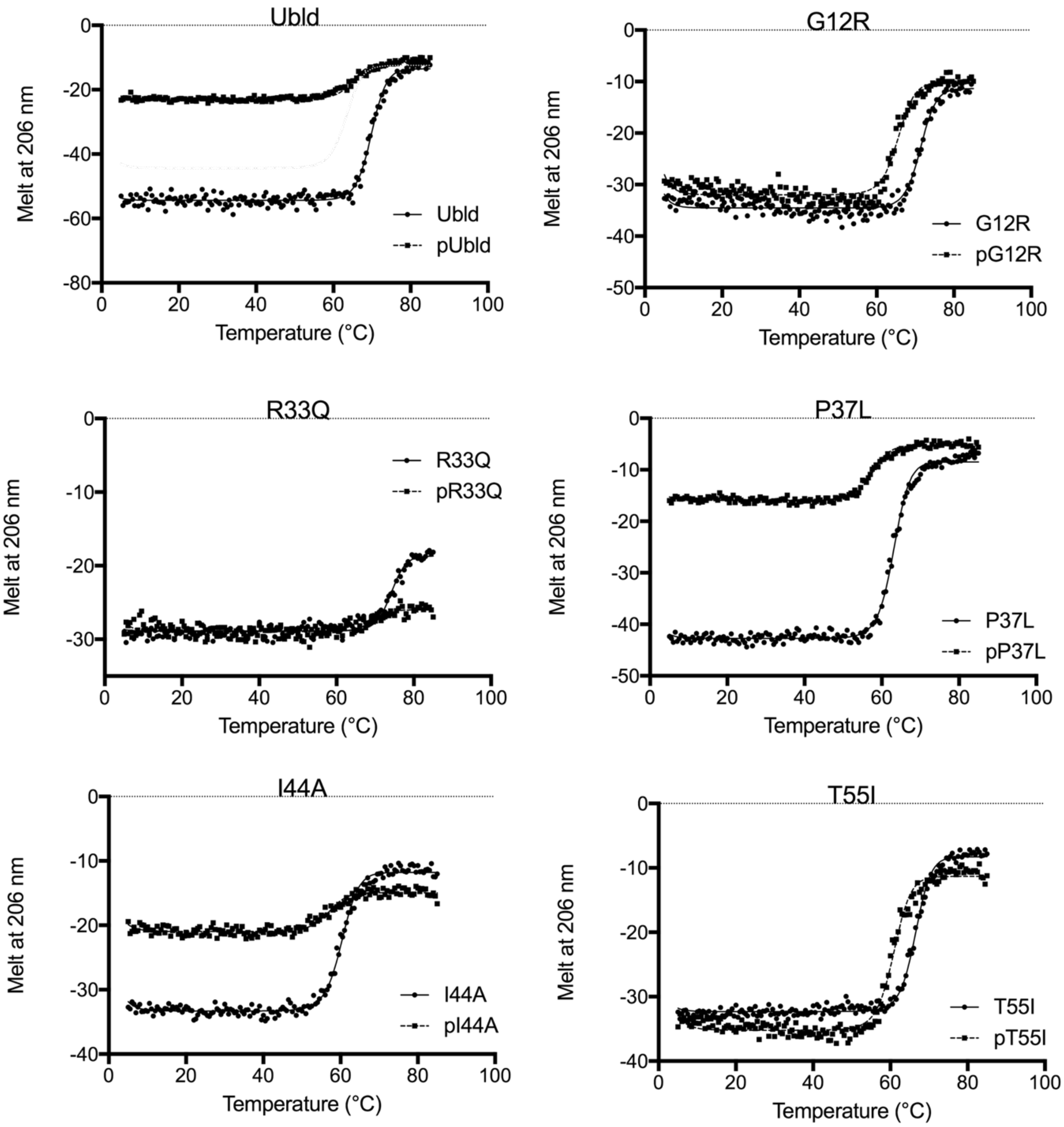

Figure S1. Thermal unfolding curves for unphosphorylated and phosphorylated parkin Ubl domain and selected ARJP substitutions. Data were collected using CD spectropolarimetry between 5-95˚C using a temperature gradient of 1 C˚/minute. The ellipticity was measured at 206 nm and fit as described in Experimental Procedures and reference (38).
