# Supplementary material for: Impact of altered phosphorylation on loss of function of juvenile Parkinsonism–associated genetic variants of the E3 ligase parkin
Source: J Biol Chem. 2018 Mar 12;293(17):6337–48. doi: 10.1074/jbc.RA117.000605 (PMC5925814; doi:10.1074/jbc.RA117.000605)
Supplement: Supporting Information [file supp_RA117.000605_133409_2_supp_89543_p56rr6.pdf]

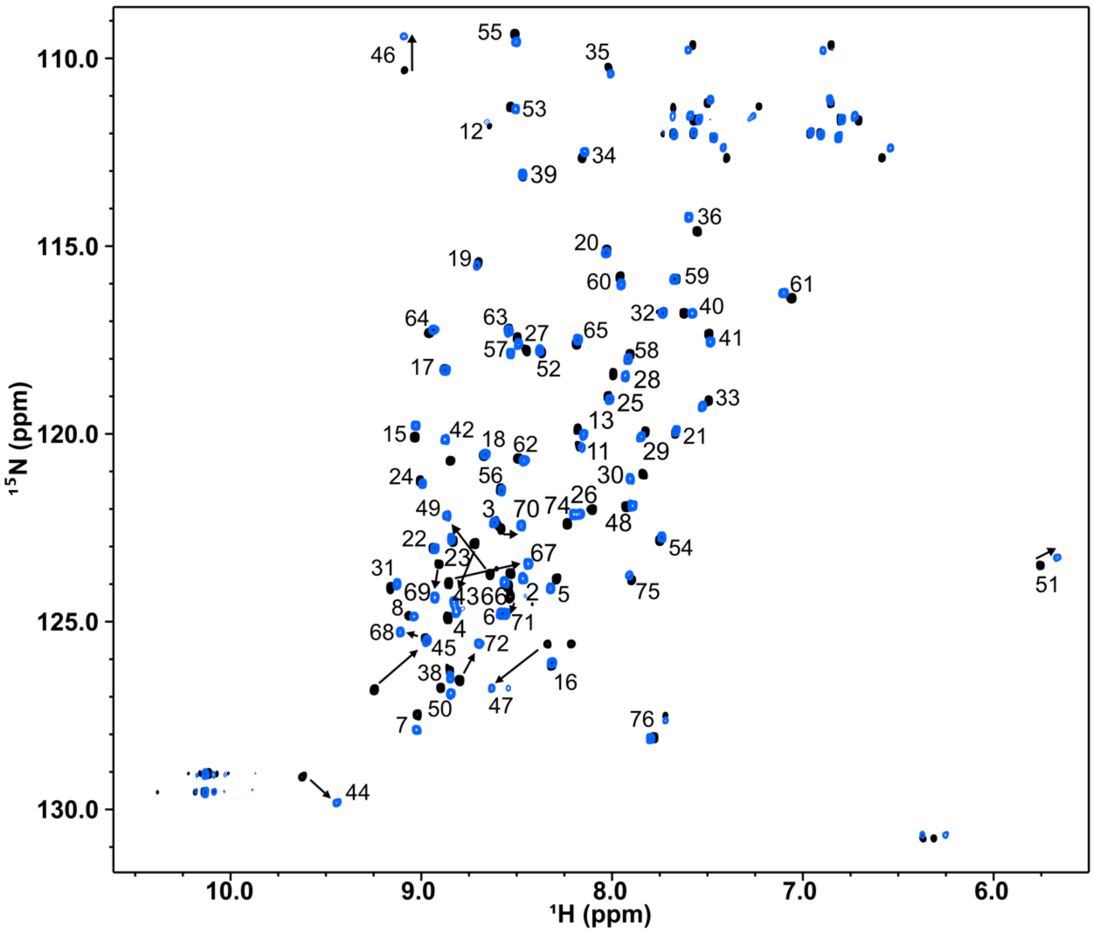

Figure S2. ^{1}H-^{15}N HSQC spectrum of parkin Ubl domain carrying an I44A substitution (Ubl^{I44A}). The Ubl^{I44A} domain spectrum (blue) is superimposed on the wild-type spectrum of the parkin Ubl domain (black). Signals that undergo the largest chemical shift changes arising from the I44A substitution are indicated by arrows.
